# Synergized Tricomponent All‐Inorganics Solid Electrolyte for Highly Stable Solid‐State Li‐Ion Batteries

**DOI:** 10.1002/advs.202207627

**Published:** 2023-07-05

**Authors:** Guixiang Xu, Xin Zhang, Shuyang Sun, Yangfan Zhou, Yongfeng Liu, Hangwang Yang, Zhenguo Huang, Fang Fang, Wenping Sun, Zijiang Hong, Mingxia Gao, Hongge Pan

**Affiliations:** ^1^ State Key Laboratory of Silicon and Advanced Semiconductor Materials Key Laboratory of Advanced Materials and Applications for Batteries of Zhejiang Province and School of Materials Science and Engineering Zhejiang University Hangzhou 310058 China; ^2^ School of Materials Science and Chemical Engineering Xi'an Technological University Xi'an 710021 China; ^3^ School of Civil & Environmental Engineering University of Technology Sydney 81 Broadway Ultimo NSW 2007 Australia; ^4^ Department of Materials Science Fudan University Shanghai 200433 China

**Keywords:** borohydrides, cyclability, garnet‐type oxides, lithium ion batteries, solid‐state electrolytes

## Abstract

Garnet‐type oxide Li_6.4_La_3_Zr_1.4_Ta_0.6_O_12_ (LLZTO) features superior ionic conductivity and good stability toward lithium (Li) metal, but requires high‐temperature sintering (≈1200 °C) that induces high fabrication cost, poor mechanical processability, and high interface resistance. Here, a novel high‐performance tricomponent composite solid electrolyte (CSE) comprising LLZTO−4LiBH_4_/*x*Li_3_BN_2_H_8_ is reported, which is prepared by ball milling the LLZTO−4LiBH_4_ mixture followed by hand milling with Li_3_BN_2_H_8_. Green pellets fabricated by heating the cold‐pressed CSE powders at 120 °C offer ultrafast room‐temperature ionic conductivity (≈1.73 × 10^−3^ S cm^−1^ at 30 °C) and ultrahigh Li‐ion transference number (≈0.9999), which enable the Li|Li symmetrical cells to cycle over 1600 h at 30 °C with only 30 mV of overpotential. Moreover, the Li|CSE|TiS_2_ full cells deliver 201 mAh g^−1^ of capacity with long cyclability. These outstanding performances are due to the low open porosity in the electrolyte pellets as well as the high intrinsic ionic conductivity and easy deformability of Li_3_BN_2_H_8_.

## Graphical abstract

Synergized tricomponent all‐inorganics solid electrolyte comprising LLZTO−4LiBH_4_/*x*Li_3_BN_2_H_8_ offers ultrafast room‐temperature ionic conductivity (≈1.73 × 10^−3^ S cm^−1^ at 30 °C) and ultrahigh Li‐ion transference number (≈0.9999), thus enabling the Li|Li symmetrical cells to cycle over 1600 h at 30 °C with only 30 mV of overpotential. Moreover, the Li|CSE|TiS_2_ full cells (CSE: composite solid electrolyte) deliver 201 mAh g^−1^ with long cyclability.

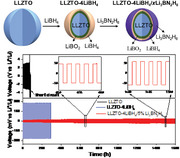

## 1 Introduction

All solid‐state lithium ion batteries (ASSLIBs) offer the great potential to meet the ever‐growing demand for electrochemical energy storage devices with higher energy density and better safety.^[^ ^1^ ^]^ Solid‐state electrolytes (SSEs) as a critical component of ASSLIBs have been extensively studied.^[^ ^2^ ^]^ The ideal SSEs must feature high ionic conductivity (>10^−4^ S cm^−1^), low electronic conductivity, high interface compatibility with electrodes, and wide voltage window (0–5 V).^[^ ^3^ ^]^ In this respect, the garnet‐type oxides have attracted intense attention because of their excellent ionic conductivity and (electro)chemical stability to Li metal.^[^ ^4^ ^]^ In particular, Ta‐doped lithium lanthanum zirconate garnet, Li_6.4_La_3_Zr_1.4_Ta_0.6_O_12_ (LLZTO) delivers a high ionic conductivity up to 10^−3^ S cm^−1^ at room temperature, comparable to the values of organic liquid electrolytes used in commercial Li‐ion batteries.^[^ ^5^, ^6^, ^7^ ^]^ With a high shear modulus (≈60 GPa), however, the garnet‐type oxides are typically stiff and very difficult to process.^[^ ^8^ ^]^ It is necessary to densify LLZTO pellets to achieve high ionic conductivity and to function as an effective separator, which typically requires high‐temperature sintering treatment (≈1200 °C, 24 h).^[^ ^9^ ^]^ Such operation not only leads to heavy Li loss but also produce a La_2_Zr_2_O_7_ impurity phase, which adversely affects ionic conductivity.^[^ ^10^ ^]^ Meanwhile, the sintered pellets are generally thick and brittle, consequently inducing poor processability.^[^ ^11^, ^12^ ^]^ Moreover, the solid–solid interfacial point contacts between LLZTO pellets and electrodes give rise to high interfacial resistance.^[^ ^13^ ^]^ All these prevent LLZTO SSEs from practical applications in ASSLIBs.

Several strategies have been attempted to address these issues, including developing advanced synthesis approaches, optimizing sintering aids, and constructing composite solid electrolytes (CSEs).^[^ ^6^, ^14^ ^]^ CSEs have been particularly effective in improving the performance.^[^ ^15^ ^]^ Considerable effort has been devoted to developing inorganic‐polymer CSE systems, including “ceramic‐in‐polymer” and “polymer‐in‐ceramic.”^[^ ^11^, ^16^, ^17^, ^18^, ^19^, ^20^ ^]^ These CSEs combine the advantages of both inorganics (high conductivity and mechanical strength) and polymers (flexibility and easy processing). Wan et al. designed a LLZTO nanowire/polyethylene oxide (PEO) CSE, which enabled all‐solid LiFePO_4_|Li full cells delivering 158.8 mAh g^−1^ after 70 cycles at 0.5 C and 60 °C.^[^ ^17^ ^]^ Similarly, Huo et al. obtained 1.6 × 10^−4^ S cm^−1^ of ionic conductivity at 30 °C by distributing 20 vol% LLZTO in the PEO polymer matrix.^[^ ^18^ ^]^ The LLZTO/poly(vinylene carbonate) fabricated by an in‐situ polymerization route displayed 7.8 × 10^−5^ S cm^−1^ of ionic conductivity at room temperature and an enlarged electrochemical window over 4.5 V (vs Li^+^/Li).^[^ ^19^ ^]^ The LLZTO/polyethylene glycol diacrylate CSE delivered a room‐temperature Li^+^ ion conductivity of 3.1 × 10^−4^ S cm^−1^ and an electrochemical polarization potential up to 4.6–4.7 V (vs Li^+^/Li).^[^ ^20^ ^]^ However, the reported Li^+^ ion transference numbers for these CSEs are usually ≈0.5, much lower than those of pristine inorganic electrolytes.^[^ ^21^ ^]^ Encouragingly, this problem was partially overcome by compositing LLZTO with LiBH_4_.^[^ ^22^ ^]^ LiBH_4_ displays several unique properties concerning SSE application, that is, low molecular weight, good compressibility, easy deformability, and high compatibility with Li metal.^[^ ^23^, ^24^ ^]^ By ball milling LLZTO−4LiBH_4_ followed by cold pressing into pellets without sintering, this CSE delivered an Li^+^ transference number as high as 0.9999, indicating great potential as a superior single‐ion conductor.^[^ ^22^ ^]^ The ionic conductivity was measured to be 8.02 × 10^−5^ S cm^−1^ at 30 °C, very close to the abovementioned inorganic‐polymer CSEs. While compared with liquid organic electrolytes used in traditional Li‐ion batteries, such ionic conductivity is lower by one to two orders of magnitude, not sufficiently high for practical applications. Therefore, it remains challenging to further elevate the room‐temperature ionic conductivity of LLZTO‐based CSEs without using complicated processing procedure.

Herein, we report a novel LLZTO‐based all‐inorganics CSE featuring ultrafast ionic conductivity and ultrahigh Li‐ion transference number at 30 °C. A synergized tricomponent CSE can be successfully obtained only by hand milling the Li_3_BN_2_H_8_ quaternary hydride with the LLZTO−4LiBH_4_ composite prepared by ball milling. Li_3_BN_2_H_8_ plays a critical role in further enhancing the room‐temperature ionic conductivity due to high intrinsic Li^+^ ion conductivity at room temperature and easy mechanical deformability.^[^ ^25^ ^]^ The resultant LLZTO−4LiBH_4_/Li_3_BN_2_H_8_ CSE pellets fabricated by cold pressing followed by heat treatment (HT) at 120 °C delivered a Li^+^ ion conductivity as high as 1.73 × 10^−3^ S cm^−1^ at 30 °C. This is nearly six orders of magnitude higher than that of the pristine LLZTO electrolyte pellets prepared under identical conditions. The Li‐ion transference number is calculated to be 0.9999 which significantly outperforms the inorganic‐polymer CSEs. In addition, the new CSE has a wide electrochemical window up to 6 V (vs Li^+^/Li). The tricomponent CSE enables the Li|Li symmetrical cells to cycle stably over 1600 h at 0.15 mA cm^−2^ and 30 °C with a quite low overpotential of 30 mV. When used in the Li|TiS_2_ full cells, a high reversible capacity of 201 mAh g^−1^ with 98.5% of retention over 80 cycles was achieved, demonstrating superior practical application potential.

## 2 Results and Discussion

### 2.1 Preparation and Characterization of Composite Solid Electrolyte

**Figure 1:**
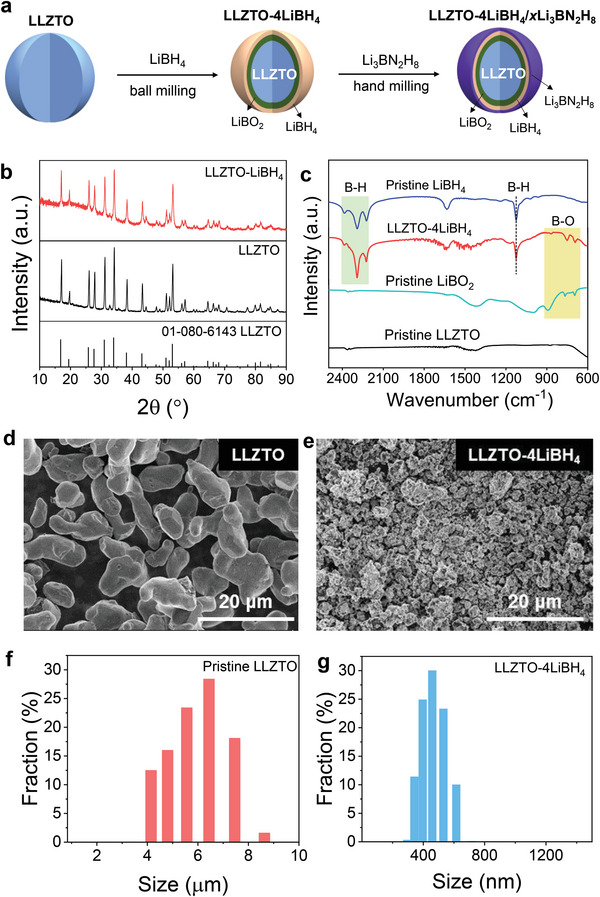
Preparation and characterization of tricomponent CSEs. a) Schematic of the preparation of tricomponent CSEs, b) XRD patterns, c) FTIR spectra, d,e) SEM images, and f,g) particle size distributions of LLZTO and LLZTO‐4LiBH_4_ (referring to L‐0).

The preparation process is schematically illustrated in **Figure** **1**a. LLZTO−4LiBH_4_ composite and quaternary hydride Li_3_BN_2_H_8_ were first prepared by ball milling the corresponding raw materials at 500 rpm for 2 and 24 h, respectively, as reported in our previous work.^[^ ^23^, ^26^ ^]^ After ball milling, the characteristic absorption peaks of B—H vibration in LiBH_4_ and B—O vibration in LiBO_2_ are clearly visible in the Fourier‐transform infrared (FTIR) spectra of LLZTO−4LiBH_4_ (Figure 1b) and the X‐ray diffraction (XRD) peaks are all associated with LLZTO (Figure 1c). Scanning electron microscope (SEM) observation displays a remarkable reduction in particle size of LLZTO with surface coating after ball milling with LiBH_4_ (Figure 1d,e). Laser particle size analysis quantitatively confirms this phenomenon because the average particle size was determined to be ≈5.9 µm for pristine LLZTO and ≈462 nm for the LiBH_4_‐containing sample (Figure 1f,g). Ball milling LiBH_4_−2LiNH_2_ induced the formation of quaternary hydride Li_3_BN_2_H_8_ composed of the *α*‐Li_4_BN_3_H_10_ primary phase along with trace Li_2_BNH_6_ impurity (Figure S1a, Supporting Information), agreeing well with the previous reports.^[^ ^27^ ^]^ Aggregation and adhesion were also observed in the resultant product (Figure S1b–d, Supporting Information).

**Figure 2:**
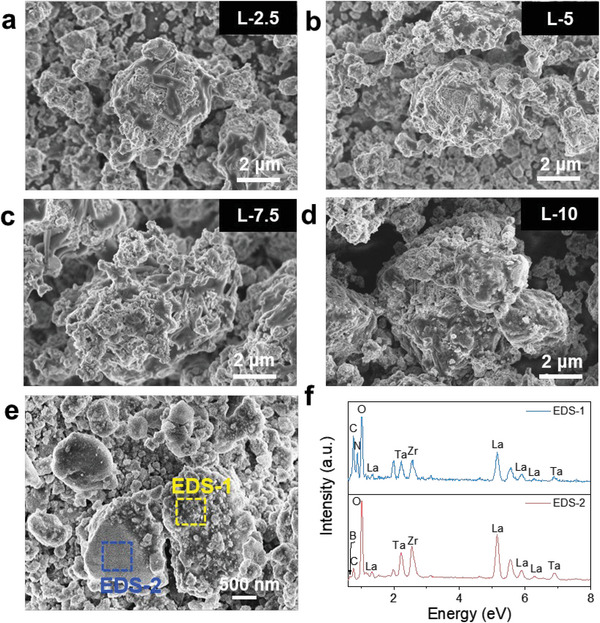
Morphology and composition investigations of tricomponent CSEs. a–d) SEM images of LLZTO−4LiBH_4_/*x*Li_3_BN_2_H_8_ samples (*x* = 2.5, 5, 7.5, and 10 wt%, referring to, L‐2.5, L‐5, L‐7.5, and L‐10, respectively), and e) SEM image and f) corresponding EDS results of L‐10.

**Figure 3:**
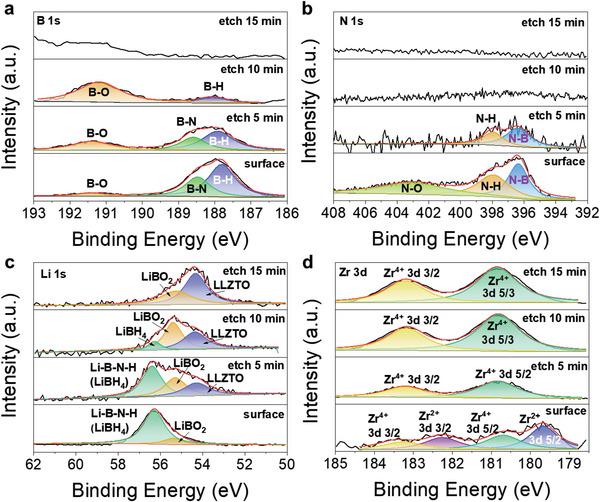
Surface composition analysis of tricomponent CSEs. XPS spectra of a) B 1s, b) N 1s, c) Li 1s, and d) Zr 3d of the L‐10 sample after etching with different times.

**Figure 4:**
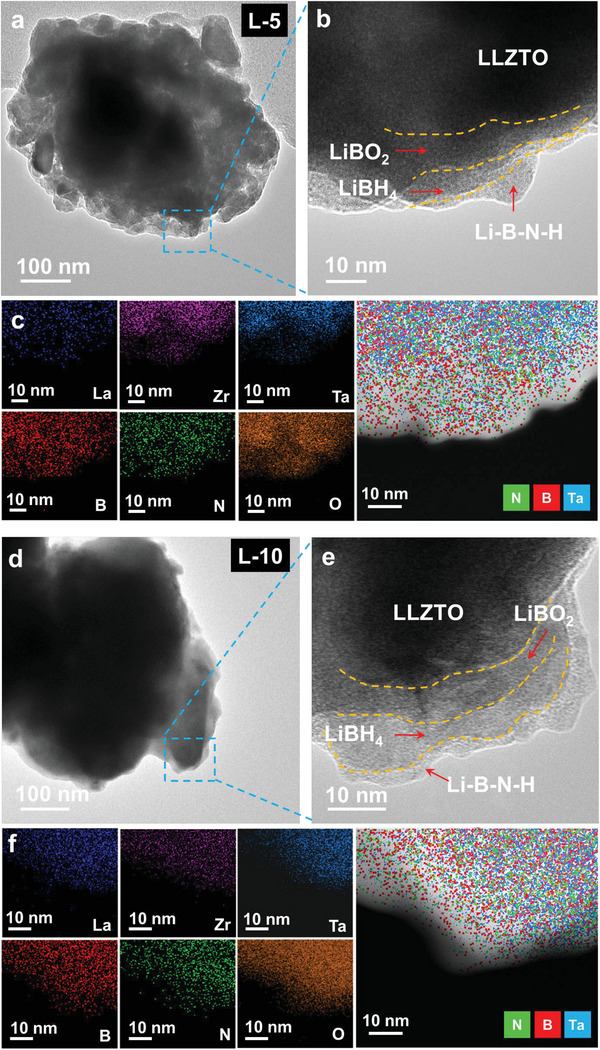
Microstructure analysis of tricomponent CSEs. a) TEM, b) HRTEM, and c) corresponding EDS element mapping of L‐5 sample, and d) TEM, e) HRTEM, and f) corresponding EDS element mapping of L‐10 sample.

Further mixing LLZTO−4LiBH_4_ with a certain amount of Li_3_BN_2_H_8_ (weight percentage: 2.5, 5, 7.5, and 10 wt%, referring to L‐2.5, L‐5, L‐7.5, and L‐10, respectively) was carried out through hand milling for 15 min. This operation gives rise to more visible surface coating as evidenced by black strips adhered to the particles (**Figure** **2**a–d). In particular, the L‐10 sample displays the encapsulation‐like morphology (Figure 2d). In contrast to other grey regions, the black strips were identified to be contained N element with energy dispersive X‐ray spectroscopy (EDS) point analyses (Figure 2e,f), indicating that they should be Li_3_BN_2_H_8_. X‐ray photoelectron spectroscopy (XPS) survey spectra also detected the N signal in the Li_3_BN_2_H_8_‐containing samples (Figure S2, Supporting Information). Further Ar^+^ ion sputtering measurement reveal a multilayer coating on the LLZTO particle surface. As shown in **Figure** **3**, the chemical states of B changed from B—H/B—N to B—H/B—O and to B—O upon sputtering, correspondingly the N‐H/N‐B for the N element disappeared gradually and the change of Li was from Li_3_BN_2_H_8_ and/or LiBH_4_ to LiBO_2_ and to LLZTO. Moreover, the low‐valent Zr^2+^ at 179.67 and 182.25 eV were detected on the surface of the resultant product (Figure S3, Supporting Information),^[^ ^28^ ^]^ implying the occurrence of reduction of some Zr^4+^ in LLZTO by reacting with LiBH_4_ as observed in FTIR results (Figure 1b). Further transmission electron microscope (TEM) observation also display a multilayer coating structure (**Figure** **4** and Figure S4, Supporting Information). In particular, the EDS mapping results reveal that the B and N signals stay on the “edge” of particles by comparing with the Ta signal as one of the representative elements in LLZTO (Figure 4c,f). By combining our previous report,^[^ ^22^ ^]^ we believe that the Li_3_BN_2_H_8_ secondary coating was successfully achieved only by hand milling Li_3_BN_2_H_8_ with the post‐milled LLZTO−4LiBH_4_, which gives rise to the formation of novel tricomponent LLZTO−4LiBH_4_/*x*Li_3_BN_2_H_8_ CSEs. However, no obvious change was observed in the XRD patterns and FTIR spectra with the presence of Li_3_BN_2_H_8_ (Figure S5, Supporting Information), possibly due to its relatively low concentration (≤10 wt%).

### 2.2 Electrochemical Performance of Composite Solid Electrolyte

**Figure 5:**
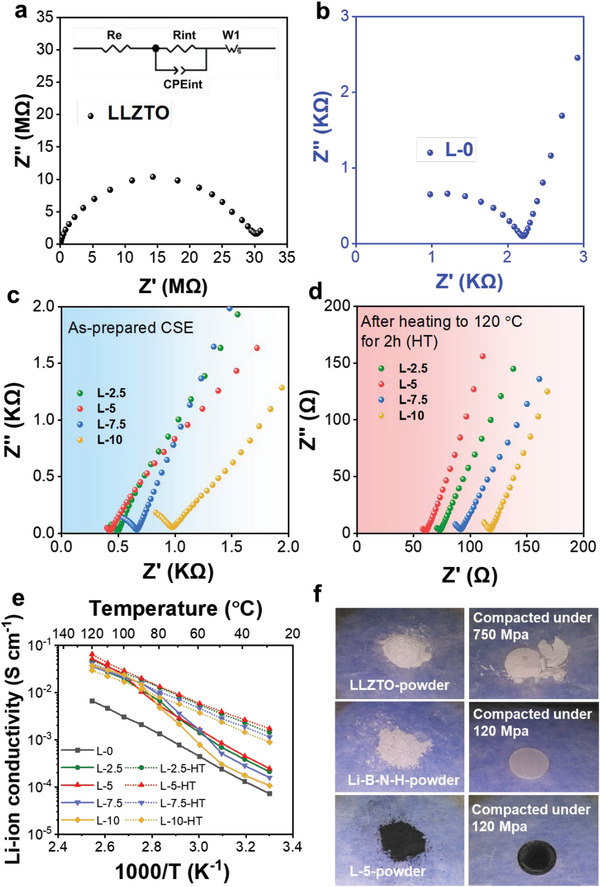
Ionic conductivity of tricomponent CSEs. a–d) Nyquist curves, e) Arrhenius plots, and f) compaction formability of the pristine LLZTO and the LLZTO−4LiBH_4_/*x*Li_3_BN_2_H_8_ CSEs before and after heat treatment.

The resultant tricomponent LLZTO−4LiBH_4_/*x*Li_3_BN_2_H_8_ CSEs were subjected to electrochemical impedance spectra (EIS) measurement by assembling SUS|SSE|SUS symmetrical cells (SUS: steel use stainless) to calculate Li^+^ ion conductivity. In stark contrast to previous reports where the LLZTO electrolyte pellets were fabricated under high pressure and high temperatures,^[^ ^29^ ^]^ our tricomponent CSE pellets were obtained only through cold pressing sample powders at room temperature, which is simple and effective. The EIS spectra were collected at 30 °C. All spectra in **Figure** **5**a–d consist of a semicircle and a straight line. It is generally accepted that the semicircle diameter is associated with resistance due to the grain boundary and pores inside the solid electrolyte (*R*_g+p_), while the straight line at low frequency region corresponds to the ion transfer resistance. The *R*_g+p_ values were determined by fitting EIS data with the equivalent circuit shown in the inset of Figure 5a and listed in **Table** **1**. The results indicate that the presence of quaternary hydride Li_3_BN_2_H_8_ largely reduced the *R*_g+p_ values of LLZTO‐based CSEs because they are all less than 1 kΩ, remarkably lower than those of the pristine LLZTO pellet (30.6 MΩ) and the L‐0 pellet (LLZTO‐4LiBH_4_, 2.2 KΩ). Further calculation using the following equation, the temperature‐depended ion conductivity (*σ*_Li+_) was obtained and plotted in Figure 5e.

(1) $$\sigma_{\mathrm{Li}+}=\frac{d}{SR_{t}}$$

Here, *S*, *d*, and *R*_t_ are the pellet area, thickness, and bulk resistance of the electrolyte pellet, respectively. It is clear that the addition of 5 wt% Li_3_BN_2_H_8_ (L‐5) gives rise to the highest ionic conductivity, especially at low temperatures. At 30 °C, the value of ionic conductivity was determined to be 3.37 × 10^−4^ S cm^−1^, which is very similar to the high‐temperature sintered LLZTO ceramic pellets reported previously (≈3.5 × 10^−4^ S cm^−1^ at 25 °C)^[^ ^29^ ^]^ but nearly five orders of magnitude higher than that of pristine LLZTO green pellets (4.03 × 10^−9^ S cm^−1^). Such high value is about fivefold higher than that of LLZTO−4LiBH_4_ (6.09 × 10^−5^ S cm^−1^). Clearly the addition of appropriate Li_3_BN_2_H_8_ improves the Li^+^ ion transport, possibly due to the intrinsic high Li^+^ ion conductivity and good mechanical deformation properties. Such speculation was evidenced by comparing the compactibility of LLZTO and Li_3_BN_2_H_8_ through cold‐pressing treatment (Figure 5f). A well‐compacted pellet was obtained while cold‐pressing Li_3_BN_2_H_8_ powders under 120 MPa of pressure, representing a good deformability and formability. Similar phenomenon was also observed for the prepared L‐5 sample, which is in stark contrast to pristine LLZTO because it is quite difficult to form a pellet even under 750 MPa of pressure. Further nanoindentation measurement presents a dramatic increase in the hardness (from 0.046 to 0.278 GPa) and the elastic modulus (from 2.506 to 6.814 GPa) for the L‐5 electrolyte pellets with respect to pristine LLZTO green pellet (Figure S6, Supporting Information). This fact reasonably indicates a good mechanical strength for the Li_3_BN_2_H_8_‐containing electrolyte green pellets.

**Table 1:** Ionic conductivity and E_a_ values of LLZTO, Li_3_BN_2_H_8_, and LLZTO−4LiBH_4_/*x*Li_4_BN_3_H_10_ (L‐0, L‐2.5, L‐5, L‐7.5, L‐10) CSEs before and after heat treatment (HT)

Samples	*R*_g+p_/Ω	*σ*_Li+_ [S cm^−1^]	*E*_a_ [eV]
LLZTO	3.06 × 10^7^	4.03 × 10^−9^	0.70
L‐0	2195	6.09 × 10^−5^	0.65
Li_3_BN_2_H_8_	2100	4.64 × 10^−5^	0.58
L‐2.5	578	1.81 × 10^−4^	0.56
L‐5	310	3.37 × 10^−4^	0.56
L‐7.5	660	1.58 × 10^−4^	0.57
L‐10	975	1.07 × 10^−4^	0.58
LLZTO‐HT	3.21 × 10^7^	4.08 × 10^−9^	0.70
L‐0‐HT	4685	3.40 × 10^−5^	0.63
Li_3_BN_2_H_8_‐HT	1308	2.23 × 10^−5^	0.61
L‐2.5‐HT	73	1.43 × 10^−3^	0.43
L‐5‐HT	60	1.74 × 10^−3^	0.43
L‐7.5‐HT	85	1.06 × 10^−3^	0.45
L‐10‐HT	117	8.93 × 10^−4^	0.47

More importantly, we observed a further remarkable reduction in the *R*_g+p_ value after heating the cold‐pressed CSE pellets at 120 °C for 2 h, indicating a much more favorable Li^+^ ion conductivity. The HT samples are denoted as L‐2.5‐HT, L‐5‐HT, L‐7.5‐HT, and L‐10‐HT, respectively, corresponding to *x* = 2.5, 5, 7.5, and 10 wt% in LLZTO−4LiBH_4_/*x*Li_3_BN_2_H_8_. The *R*_g+p_ values were further decreased to below 100 Ω (the bottom of Figure 5d) and accordingly the Li^+^ ion conductivities were elevated by about one order of magnitude up to 1.74 × 10^−3^ S cm^−1^ for the 5 wt% Li_3_BN_2_H_8_−containing sample (L‐5‐HT) (Figure 5e). This Li^+^ ion conductivity value is also about two orders of magnitude higher than that of LLZTO−4LiBH_4_ (6.09 × 10^−5^ S cm^−1^), and even reaches the level of the organic liquid electrolyte (≈10^−3^ S cm^−1^).^[^ ^30^ ^]^

### 2.3 Mechanism for Enhanced Ionic Conductivity

**Figure 6:**
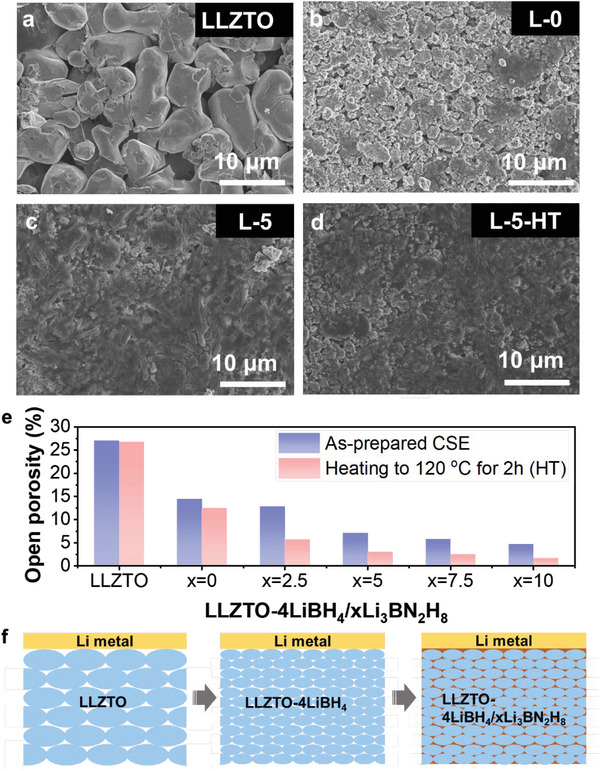
Green pellet morphology and structure characteristics. SEM images of a) LLZTO, b) L‐0, and L‐5 pellets c) before and d) after heat treatment. e) Open porosity and f) illustrative microstructures of the cold pressed SSEs.

To understand the reason for the enhanced ionic conductivity, the apparent activation energy (*E*_a_) for Li^+^ ion transport was calculated and compared by fitting the temperature dependence data at 30–120 °C shown in Figure 5e using the Arrhenius equation. The results are listed in Table 1. The relative lower *E*_a_ values were obtained for the tricomponent LLZTO−4LiBH_4_/*x*Li_3_BN_2_H_8_ samples, especially for the 120 °C‐heated samples. The *E*_a_ value of L‐5‐HT sample was determined to be only 0.43 eV, lowering by 30% with respect to the LLZTO−4LiBH_4_ sample (0.63 eV), indicating a much reduced kinetic energy barrier for Li^+^ ion transport. This is presumably related to the densification of the electrolyte pellets and the intrinsic high ionic conductivity of additional Li_3_BN_2_H_8_.^[^ ^24^ ^]^ Such conjecture was well evidenced by SEM observation. As shown in **Figure** **6**a–d, it is clearly observed that the presence of Li_3_BN_2_H_8_ remarkably reduced the cavities and holes on the surface and bulk of electrolyte pellets. In particular, the HT at 120 °C gave rise to a much more compacted pellet with respect to the green pellet (Figure 6c,d), while no appreciable change was observed in the XRD profiles (Figure S7, Supporting Information), indicating a relatively stable phase structure. The high density of the electrolyte pellets was also indicated quantitatively by the open porosity measurement. As shown in Figure 6e, the presence of Li_3_BN_2_H_8_ effectively reduces the open porosity from 14.5% for *x* = 0 (L‐0) to 7.1% for the *x* = 5% composite (L‐5) and further to 4.7% for the *x* = 10% composite (L‐10). A further decrease in the open porosity was observed after HT at 120 °C for 2 h. The open porosity was measured to be only 3% for the L‐5‐HT sample, indicating ≈97% of density.

The remarkably enhanced mechanical properties are mainly attributed to two factors. On one hand, this is related to the reduced particle size of the resultant product as well as the good ductility and mechanical deformation of LiBH_4_ and Li_3_BN_2_H_8_, as mentioned in Figures 1d–g and 5f, respectively. On the other hand, the low melting point of Li_2_BNH_6_ impurity in quaternary hydride Li_3_BN_2_H_8_ also plays an important role. The remarkable melting peak was detected at ≈95 °C while heating the L‐5 sample from room temperature to 120 °C (Figure S8, Supporting Information). The melted Li_2_BNH_6_ helped Li_3_BN_2_H_8_ to fill the pores of composite pellets, as schematically indicated in Figure 6f. The highly dense pellets largely strengthened the physical contact between the CSEs’ particles, which is particularly favorable for the Li^+^ ion transfer between different particles. Considering the intrinsic ionic conductivity of pristine LLZTO (≈10^−3^ S cm^−1^), LiBH_4_ (<10^−6^ S cm^−1^), and Li_3_BN_2_H_8_ (≈10^−4^ S cm^−1^) at room temperature,^[^ ^25^ ^]^ we believe that a body‐phase Li^+^ ion transfer mechanism is much more probable for the tricomponent CSEs developed in this work than the interface transport because of the limitation of Li^+^ transfer in the surface coating layer of LiBH_4_ and Li‐B‐N‐H. Such speculation was evidenced by calculating theoretically the energy barrier of Li‐ion migration at the interface between LLZO and LiBH_4_ or Li_4_BN_3_H_10_ (Figure S9, Supporting Information). For the interface between LLZO and LiBH_4_, the energy barrier of in‐plane migration (2.240 eV at most) is larger than the out‐of‐plane migration from LLZO to LiBH_4_ (1.483 eV), indicating that Li‐ions are easier to diffuse across than along the interface. Furthermore, the energy barriers of Li‐ion migration for LLZO‐Li_4_BN_3_H_10_ interface are lower than that for LLZO‐LiBH_4_ interface, both in‐plane (1.065 eV) and out‐of‐plane (0.796 eV), representing that the Li‐ions migrate more easily at LLZO‐Li_4_BN_3_H_10_ interface than LLZO‐LiBH_4_ interface. More importantly, the energy barrier for Li‐ion migration from LiBH_4_ or Li_4_BN_3_H_10_ to LLZO is much lower than along the opposite direction, which indicates that the Li‐ions are more likely to diffuse from lithium borohydride to LLZO.

Moreover, it is worth mentioning that Li_2_BNH_6_ transformed into Li_4_BN_3_H_10_ after heating to 120 °C and then cooling down to room temperature (Figure S10a, Supporting Information). Note that the ionic conductivity for Li_4_BN_3_H_10_ at room temperature is higher than that for Li_2_BNH_6_ (Figure S10b, Supporting Information), related to a different Li^+^ arrangement in their structures (Figure S11, Supporting Information).^[^ ^31^ ^]^ These factors work together to largely facilitate the Li^+^ ion transport in the bulk electrolyte pellets fabricated by cold pressing followed by HT at 120 °C. However, too much Li_3_BN_2_H_8_ gives rise to a thicker coating on the particle surface, which retarded to some extent the Li^+^ ion transport between the particles of composites, further verifying the body‐phase transfer mechanism. As a result, the L‐5‐HT sample offered the optimal Li^+^ ion conductivity in the present study.

### 2.4 Electrochemical and Chemical Stability of Composite Solid Electrolyte

**Figure 7:**
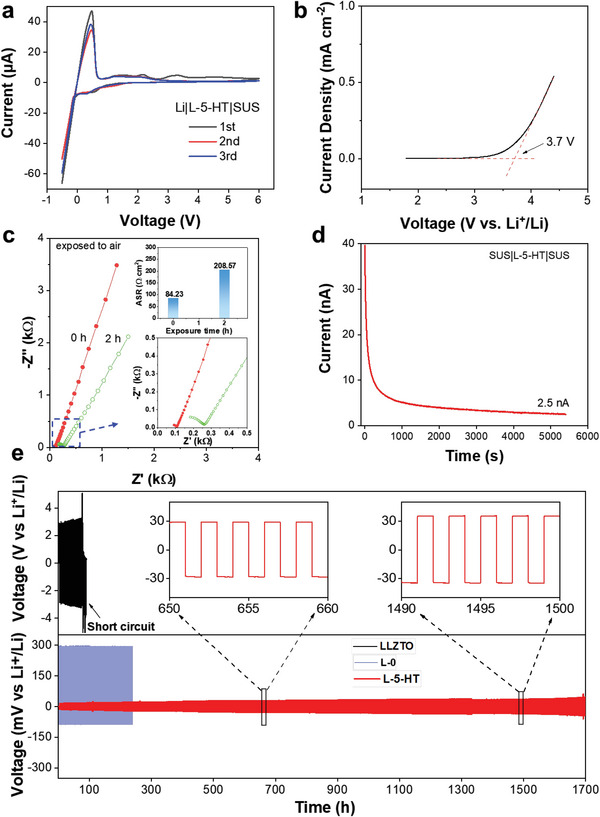
Electrochemical performance of symmetrical cells. a) CV curves, b) LSV, c) Nyquist curves and enlarged view (the bottom inset) and ASR (the upper inset) L‐5‐HT electrolyte after exposing to air, and d) DC polarization curve of the electrochemical cells assembled with L‐5‐HT electrolyte at 30 °C. e) Galvanostatic cycling curves at 30 °C of the electrochemical cells assembled with LLZTO, L‐0, and L‐5‐HT electrolyte. The insets show the detailed voltage plateau of Li stripping/plating at selected cycles.

Electrochemical stability of the Li_3_BN_2_H_8_‐containing tricomponent CSEs against Li metal was evaluated by cyclic voltammetry (CV) examination. The Li|L‐5‐HT|SUS batteries were assembled at room temperature. The sweep speed is 1 mV s^−1^ and the voltage range is −0.5 to 6 V. It is observed that from **Figure** **7**a, in addition to a strong Li stripping peak at 3.7 V, there are several small oxidation peaks in the first anodic scan, which are invisible in the following cathodic scan. These irreversible reactions between CSEs and Li metal are associated with the formation of solid electrolyte interphase films, which leads to stable cyclability since they can prevent further side reaction. Linear sweep voltammetry (LSV) was conducted from 3 to 6 V at a scan rate of 10 mV s^−1^. As shown in Figure 7b, no obvious anodic current until 3.7 V for the L‐5‐HT sample was seen, confirming a relatively wide electrochemical window. However, it should be mentioned that an obvious increase in the area specific resistance (ASR) was observed from 84.23 to 208.57 Ω cm^2^ after 2 h of exposure to standard air (Figure 7c), revealing a relatively poor chemical stability with the presence of metal hydrides. Fortunately, this problem can be successfully tackled by introducing a minor of polyimide (PI) which leads to very similar EIS spectra even after 24 h of exposure as indicated in Figure S12, Supporting Information. This is mainly attributed to the good protection by PI because hand milling the mixture of L‐5 and PI for 30 min induced the coating of PI on the surface of L‐5 sample particles (Figure S13, Supporting Information) without chemical changes (Figure S14, Supporting Information).

**Figure 8:**
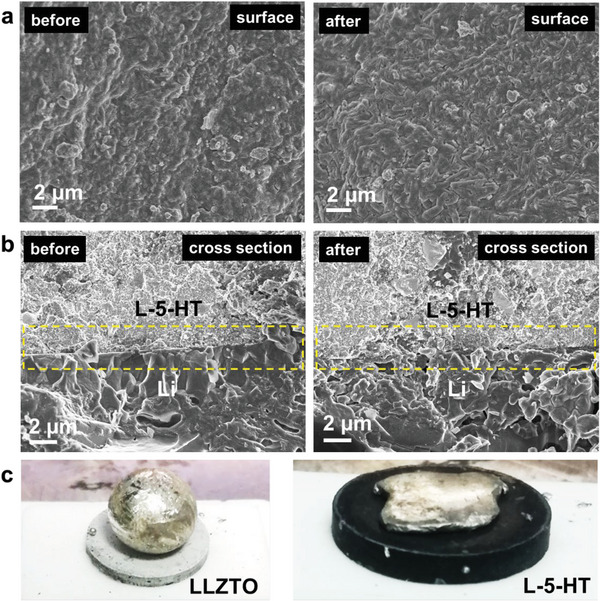
Morphology observation of L‐5‐HT electrolytes before and after cycling test. SEM images of a) surface and b) cross section of L‐5‐HT electrolytes before and after cycling test. c) Wettability test of melted Li on LLZTO and L‐5‐HT electrolytes.

The electronic conductivity was further measured by conducting direct current (DC) polarization on SUS|L‐5‐HT|SUS block cells at 30 °C. The results are shown in Figure 7d. After applying a step voltage of 100 mV, the discharge current was first rapidly and then sluggishly decreased to a steady state. The steady current should originate from the electron migration, which was measured to be ≈2.5 nA. Accordingly, the electronic conductivity was calculated to be 2.06 × 10^−9^ S cm^−1^, and the Li^+^ transference number was determined to be over 0.9999. The electronic conductivity of the L‐5‐HT sample is nearly negligible, which is of great significance for inhibiting the growth of Li dendrites and improving the cycling performance because high electronic conductivity was disclosed to be the primary origin of lithium dendrite formation within solid electrolytes.^[^ ^32^ ^]^ Indeed, the galvanostatic charge–discharge results present a remarkable long‐term cyclability for the L‐5‐HT sample, which stably cycled over 1600 h with only ≈30 mV of overpotential at 0.15 mA cm^−2^ and 30 °C (Figure 7e). Such cycling performance is largely superior to the LLZTO and LLZTO−4LiBH_4_ samples, which are possibly due to better interface contact as well as compatibility and wettability toward Li metal. On one hand, SEM observation presents a flat and dense surface morphology for the L‐5‐HT electrolyte pellet even after cycling for 1600 h (**Figure** **8**a), and the cross‐sectional images display a good interface contact between CSE and Li metal electrode without the visible fissures before and after cycling (Figure 8b). On the other hand, a remarkably improved wettability was also obtained for the Li_3_BN_2_H_8_‐containing electrolyte because the molten Li metal well spreads over the pellet, while it adopts a ball‐like morphology on top of the pristine LLZTO pellet (Figure 8c). Further EIS measurements (Figure S15, Supporting Information) revealed a slight increase in the ASR from 80.7 Ω cm^2^ at the first cycle to 91 Ω cm^2^ after 200 h, consequently ruling out the occurrence of soft short circuit upon cycling. Moreover, it should be noted that the varied current density measurement revealed 1.3 mA cm^−2^ of critical current density (CCD, Figure 8d), much higher than those of LLZTO (<0.3 mA cm^−2^) and LLZTO−4LiBH_4_ (≈0.7 mA cm^−2^).^[^ ^22^ ^]^ All these prove that Li_3_BN_2_H_8_ significantly facilitates the large‐current charge–discharge performance.

### 2.5 Full Cell Performance of Composite Solid Electrolyte

**Figure 9:**
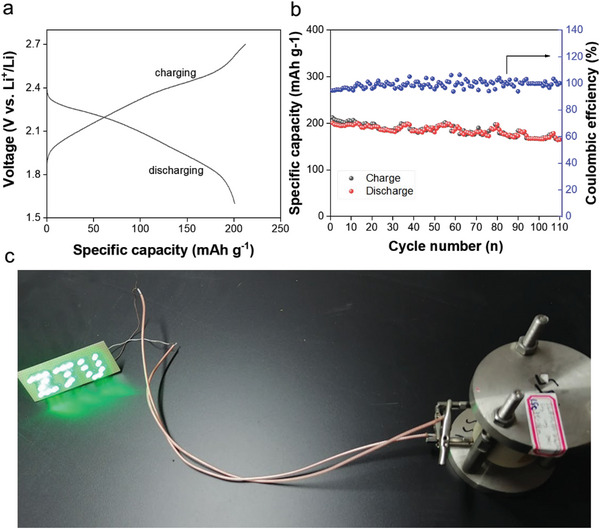
Electrochemical performance of full cells. a) First charge/discharge profile and b) cycling performance of a TiS_2_|L‐5‐HT|Li full cell. c) Digital photograph of LED array powered by the full cell.

To test the applicability of the tricomponent CSEs, we assembled full cells with TiS_2_ as the cathode, Li metal as the anode, and L‐5‐HT as the electrolyte. **Figure** **9** shows the first charge/discharge potential curves and the cycling stability of Li|L‐5‐HT|TiS_2_. The full cell delivers ≈201 mAh g^−1^ of discharge specific capacity with 94.6% of initial Coulombic efficiency while cycled at 0.1C (Figure 9a). After 80 cycles the specific capacity is still above 198 mAh g^−1^, corresponding to 98.5% of capacity retention (Figure 9b). The fully charged Li|L‐5‐HT|TiS_2_ cell successfully powered 22 green LED lights operated at 2 V and 5 mA (Figure 9c). Moreover, a preliminary success was also attained to run a full cell assembled with a LiNbO_3_‐coated LiCoO_2_ as the cathode (Figure S16, Supporting Information). All these results indicate high potential for practical applications.

## 3 Conclusion

In summary, we successfully demonstrated a novel synergized tricomponent CSE LLZTO−4LiBH_4_/*x*Li_3_BN_2_H_8_, which was prepared by first ball milling LLZTO with LiBH_4_ and then hand milling with Li_3_BN_2_H_8_. With low molecular weight, good compressibility, easy deformability, and high compatibility with Li metal, the concurrence of LiBH_4_ and Li_3_BN_2_H_8_ promotes the densification of the cold‐pressed green pellets by working as a filler, binder, and ion conducting bridge. More importantly, the high intrinsic Li^+^ ion conductivity of Li_3_BN_2_H_8_ at room temperature facilitates Li^+^ ion transport across the interface between LLZTO particles by forming continuous ionic conductive networks. Therefore, the resultant all‐inorganics CSE displays ultrafast room‐temperature Li^+^ conductivity, and ultrahigh Li‐ion transference number. The Li^+^ conductivity of L‐5‐HT sample is 1.73 × 10^−3^ S cm^−1^ at 30 °C, and the Li^+^ transference number is 0.9999. The Li|Li symmetrical cells fabricated with L‐5‐HT as the electrolyte cycled stably over 1600 h with only ≈30 mV of overpotential at 0.15 mA cm^−2^ and 30 °C. The Li|L‐5‐HT|TiS_2_ full cell delivers 201 mAh g^−1^ of discharge specific capacity with 94.6% of initial Coulombic efficiency while cycled at 0.1C, with 98.5% of capacity retention after 80 cycles.

## 4 Experimental Section

### Materials Synthesis

Raw materials including LLZTO (purity: 99.99%, MTI), LiBH_4_ (purity: 95%, Acros), and LiNH_2_ (purity: 95%, Alfa) were purchased. The LLZTO−4LiBH_4_ mixture and the quaternary hydride Li_3_BN_2_H_8_ were first prepared respectively by a simple mechanical milling process. The LiBH_4_‐LiNH_2_ mixture at a mole ratio of 1:2 was used as the starting materials for Li_3_BN_2_H_8_. Mechanical milling was conducted on a QM‐3SP4 planetary ball mill (Nanjing) at 500 rpm. The ball‐to‐sample weight ratio was set to 120:1 and the milling duration was 2 h for LLZTO‐4LiBH_4_ and 4 h for Li_3_BN_2_H_8_, as reported previously.^[^ ^22^, ^26^ ^]^ The final composite electrolytes with compositions of LLZTO−4LiBH_4_/*x*Li_4_BN_3_H_10_ (*x* = 0, 2.5%, 5%, 7.5%, 10%) were obtained by hand milling the mixtures of LLZTO−4LiBH_4_ and Li_3_BN_2_H_8_ for 15 min, which were denoted as L‐0, L‐2.5, L‐5, L‐7.5, and L‐10, respectively. All sample handlings were performed inside an Ar‐filled glove box (MBRAUN, Germany).

### Structural Characterization

The crystal structures of LLZTO‐4LiBH_4_/*x*Li_3_BN_2_H_8_ composite electrolytes were characterized on a Rigaku MiniFlex 600 XRD with Cu K_*α*_ radiation (*λ* = 0.154056 nm) operating at 40 kV and 15 mA. Data collection was performed in 2*θ* angle range of 10–90° with a 0.02° increment. A Bruker Tensor 27 unit (Germany) was used to record FTIR spectra. The powdery sample was grinded with KBr at a mass ratio of 1:300, and then cold pressed into a pellet for measurement. The morphology observation was conducted on SEM (Hitachi SU8010) operating at 3 kV, and a FEI Tecnai G2 F20 S‐TWIN TEM operating at 200 kV. The open porosity of cold‐pressed SSE pellets was measured in *n*‐heptane medium (Sinopharms). Before immersion into *n*‐heptane, the outgas operation was carried out by vacuuming in a suction flask. Thermal analysis was performed by differential scanning calorimetry (DSC) on a Netzsch DSC 200 F3 unit. Approximately 2 mg of sample was heated in an Al_2_O_3_ crucible from room temperature to 120 °C at 2 °C min^−1^ and then cooled down. XPS analyses were conducted on an ESCALAB 250 Xi spectrometer with Al K_*α*_ X‐ray source (*λ* = 0.83401 nm) under a base pressure of 5 × 10^−10^ Torr. The sample was first cold pressed into a pellet inside an argon‐filled glove box and then mounted on a sample holder which was transferred using a special container from the glove box to the XPS facility to avoid air exposure. The Ar^+^ ion sputtering was operated at 2000 eV (400 µm spot size) with a sputtering area of 2.5 mm × 2.5 mm to obtain the XPS depth profiles. The interfacial wettability was examined by dropping molten Li on the electrolyte pellets at 25 °C in an argon‐filled glove box. The Bruker Hysitron TI980 nanoindentation system (Germany) equipped with a standard berkovich indenter was used to investigate the mechanical properties of the electrolyte pellets. The LLZTO and L‐5 powder were first compacted into pellets under 300 MPa before testing. During the testing, the displacement of indenter was used to control the loading process. The typical testing process included loading–holding–unloading processes, with a duration of 5, 2, and 5 s respectively. The hardness and Young's modulus were calculated by using the load‐displacement curves.

### Electrochemical Measurements

The test cells were assembled by using SUS or Li metal electrodes to sandwich the SSE, which were put into a polyether ether ketone cylinder.^[^ ^26a^ ^]^ The SSEs were first cold pressed into pellets with a diameter of 10 mm and a thickness of 0.7–1.5 mm before the assembly of cells. EIS analysis was measured on SUS|SSE|SUS cells with an Ivium Vertex electrochemical workstation (the Netherlands) in the frequency range of 1 MHz–0.1 Hz. A constant voltage of 100 mV was applied to conduct DC polarization for 2 h. CV measurement was carried out on Li|SSE|SUS cells from −0.5 to 6 V at a scan rate of 1 mV s^−1^. The galvanostatic plating–stripping cycling was performed on Li|SSE|Li symmetric cells using Neware battery test systems (CT‐3008W‐5V20A‐S4, Shenzhen, China). The CCD was determined by cycling at elevated current densities. The TiS_2_|SSE|Li full cells were assembled and tested from 1.6 to 2.8 V. The cathode powders were prepared by mixing TiS_2_ with SSE at a weight ratio of 2:3.

### Theoretical Calculations

First‐principal calculation were performed by the Vienna Ab initio Simulation Package DFT calculation (VASP)^[^ ^33^ ^]^ based on plane‐wave DFT with the projector argument wave^[^ ^34^ ^]^ methods. The local generalized gradient approximation of Perdew, Burke, and Emzerhof^[^ ^35^ ^]^ functional was implemented. A plane wave energy cutoff of 550 eV was used. The crystal structure of cubic Li_7_La_3_Zr_2_O_12_(c‐LLZO), LiBH_4_, and Li_4_BN_3_H_10_ was fully relaxed. The c‐LLZO (110), LiBH_4_ (002), and Li_4_BN_3_H_10_ (110) surfaces were used to build interface between LLZO and LiBH_4_, LLZO, and Li_4_BN_3_H_10_, as they were low‐energy surfaces.^[^ ^36^ ^]^ LLZO (110) slab was matched with 3 × 2 × 1 super cell of LiBH_4_ (002) and 1 × 1 × 1 Li_4_BN_3_H_10_ (110) slab, with lattice mismatches less than 6%. A vacuum region of 20 Å was introduced to avoid any spurious interactions between the periodic replica. All the interface structures were then fully relaxed. The climbing‐image nudged elastic band method using VASP Transition State Tools^[^ ^37^ ^]^ coded VASP. In consideration of computing accuracy and spent, the convergency criterion was set to be the energy of 10^−4^ eV for electronic steps and the force of 0.03 eV Å^−1^ for ionic steps, and gamma‐only k‐point mesh was used. Both in‐plane and out‐of‐plane migration pathway of Li ion in the interface was calculated.

## Conflict of Interest

The authors declare no conflict of interest.

## Supporting information

Supporting Information

## Data Availability

The data that support the findings of this study are available from the corresponding author upon reasonable request.
