# Supplementary material for: Synergized Tricomponent All‐Inorganics Solid Electrolyte for Highly Stable Solid‐State Li‐Ion Batteries
Source: Adv Sci (Weinh). 2023 Jul 5;10(25):2207627. doi: 10.1002/advs.202207627 (PMC10477850; doi:10.1002/advs.202207627)
Supplement: Supplementary file 1 — Supporting Information [file ADVS-10-2207627-s001.pdf]

ADVANCED SCIENCE
Open Access

Supporting Information

for *Adv. Sci.*, DOI 10.1002/advs.202207627

Synergized Tricomponent All-Inorganics Solid Electrolyte for Highly Stable Solid-State Li-Ion Batteries

Guixiang Xu, Xin Zhang, Shuyang Sun, Yangfan Zhou, Yongfeng Liu, Hangwang Yang, Zhenguo Huang, Fang Fang*, Wenping Sun, Zijiang Hong, Mingxia Gao and Hongge Pan**

Supporting Information

Synergized Tricomponent All-inorganics Solid Electrolyte for Highly Stable Solid-state Li-ion Batteries

Guixiang Xu, Xin Zhang, Shuyang Sun, Yangfan Zhou, Yongfeng Liu, Hangwang Yang, Zhenguo Huang, Fang Fang*, Wenping Sun, Zijian Hong, Mingxia Gao, and Hongge Pan**

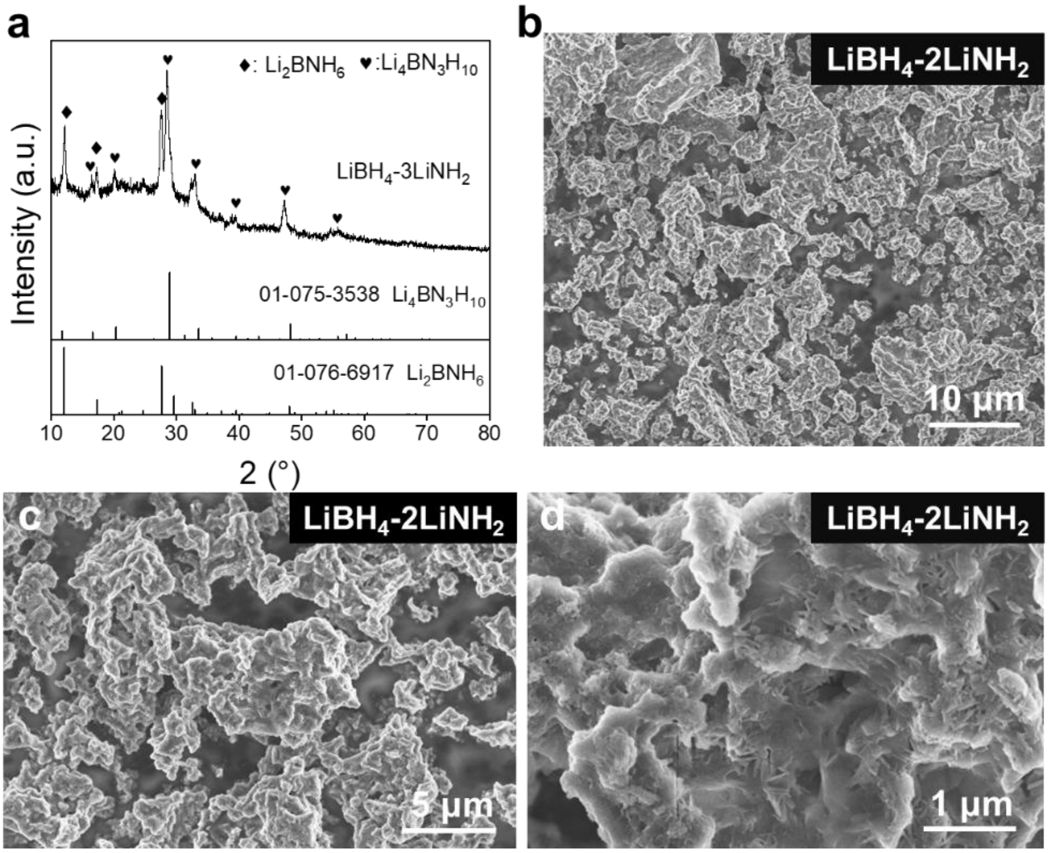

Figure S1. (a) XRD pattern and (b-d) SEM images of the prepared $Li_3BN_2H_8$ ($LiBH_4$-$2LiNH_2$) samples.

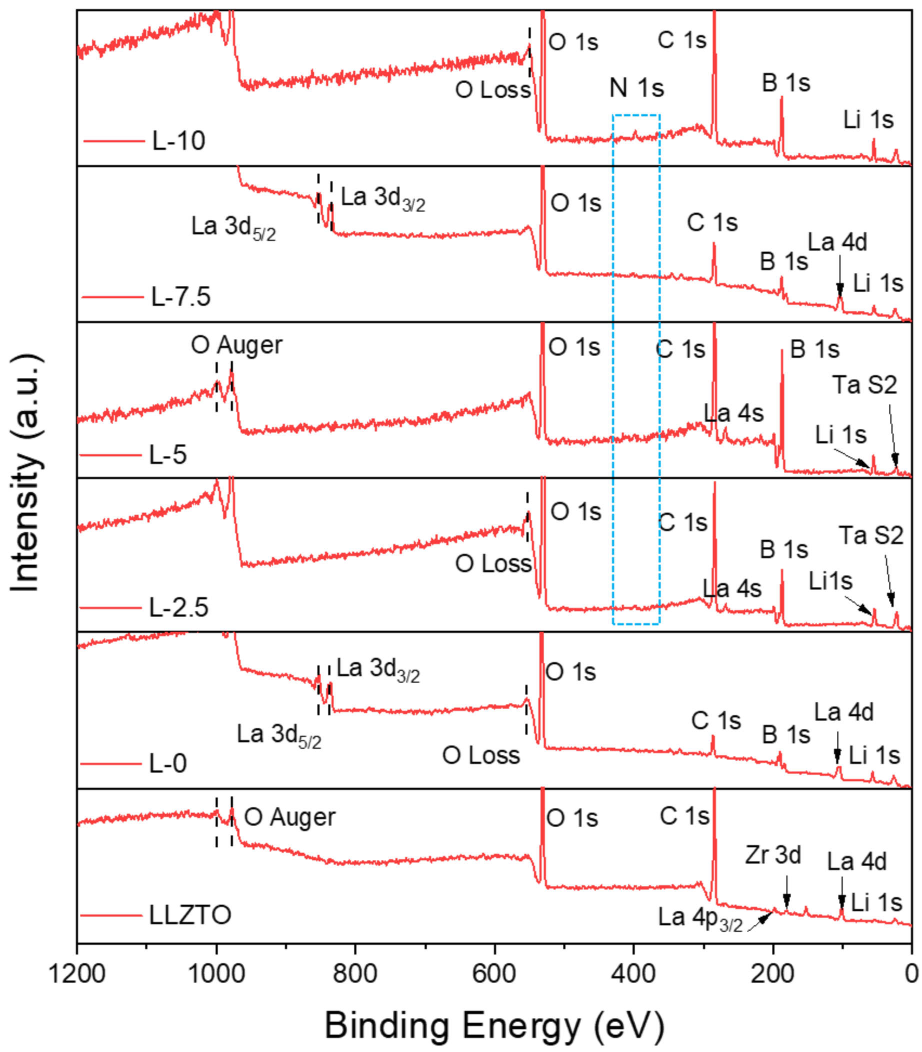

Figure S2. XPS survey spectra of LLZTO and L-0 – L-10 samples.

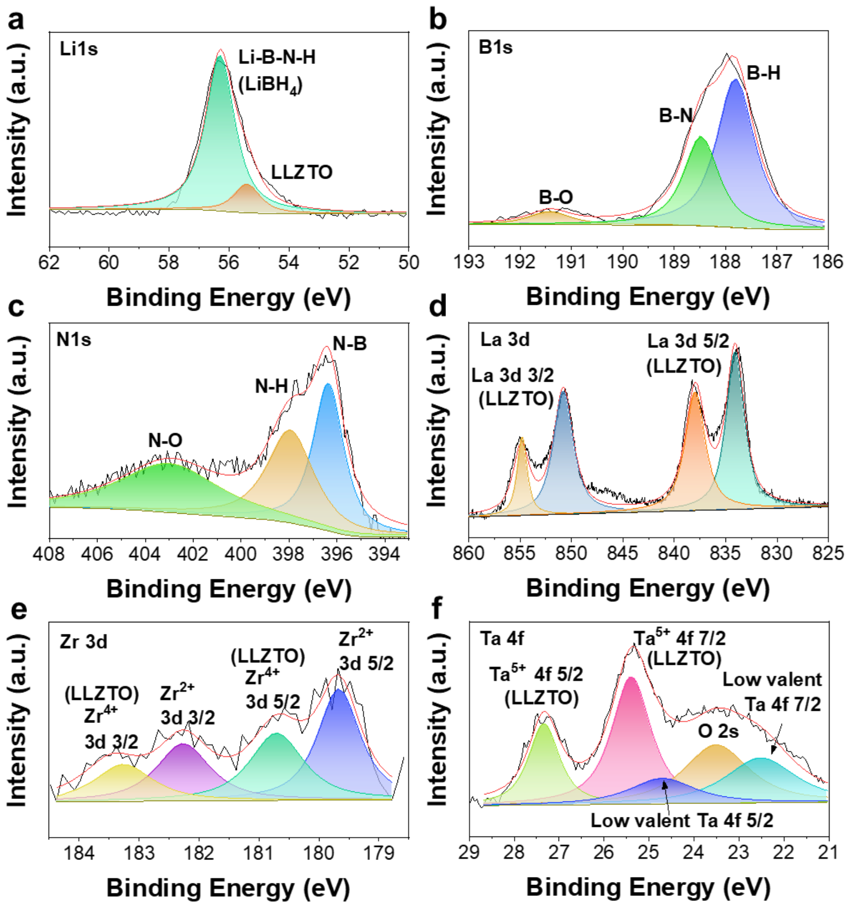

Figure S3. High-resolution XPS spectra of (a) Li 1s, (b) B 1s, (c) N 1s, (d) La 3d, (e) Zr 3d and (f) Ta 4f in L-10 sample.

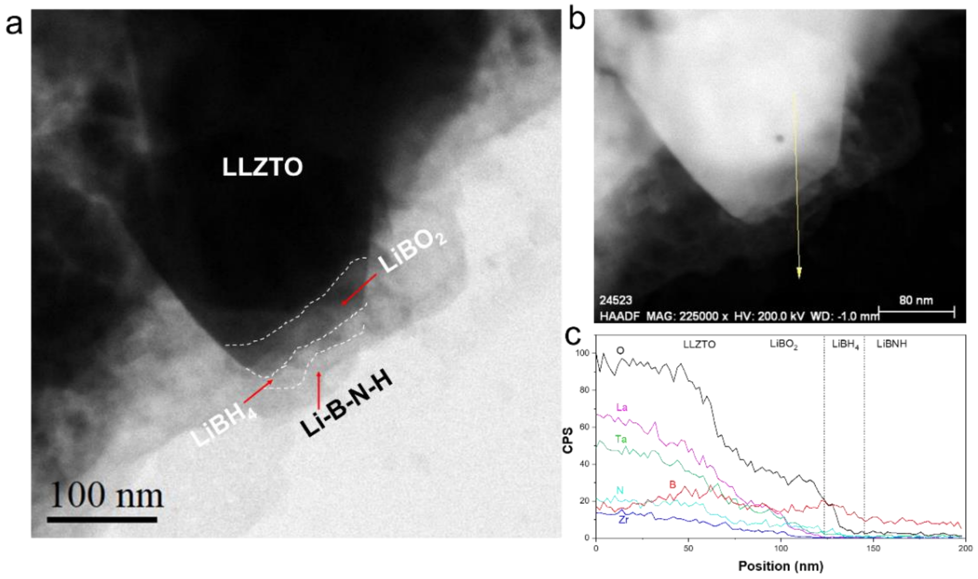

Figure S4. (a) STEM images, and (b) corresponding EDS linear analyses of the prepared LLZTO-4$LiBH_4$/$Li_3BN_2H_8$ sample.

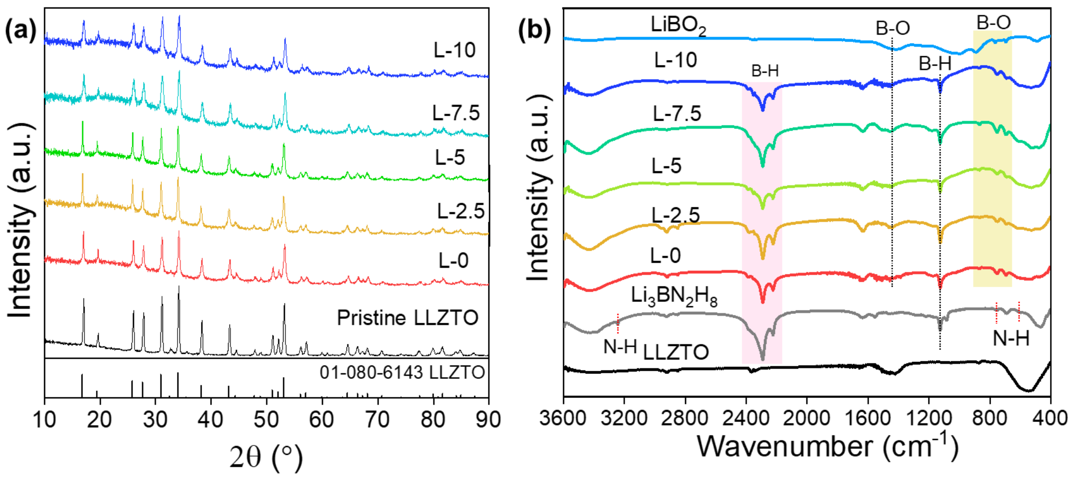

Figure S5. (a) XRD patterns and (b) FTIR spectra of different samples.

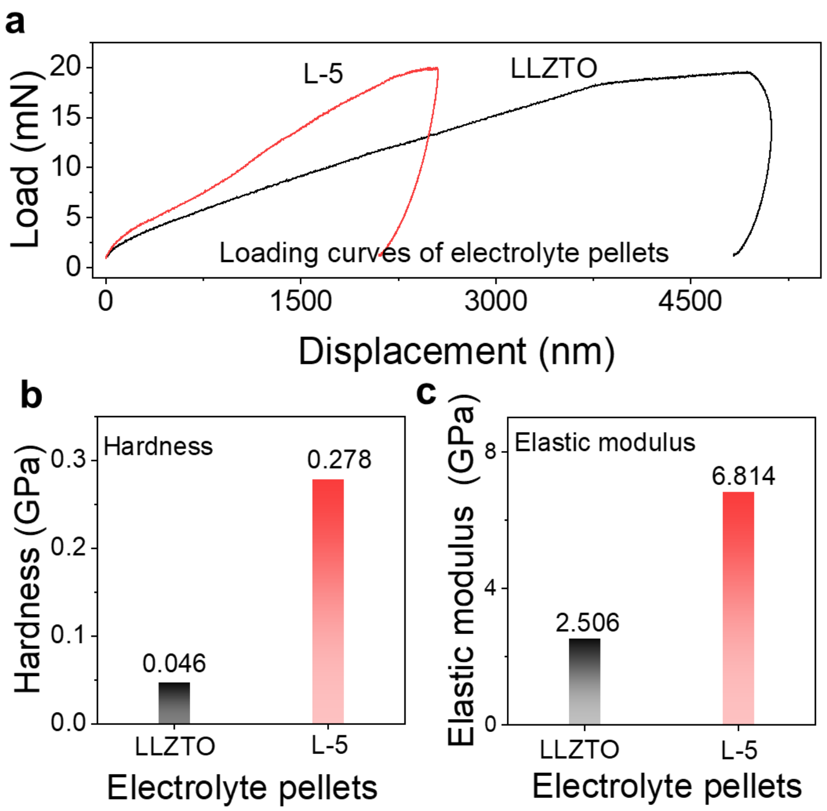

Figure S6 (a) Loading curve of nanoindentation tests on LLZTO and L-5 pellets, (b) Hardness and (c) Elastic modulus of LLZTO and L-5 pellets.

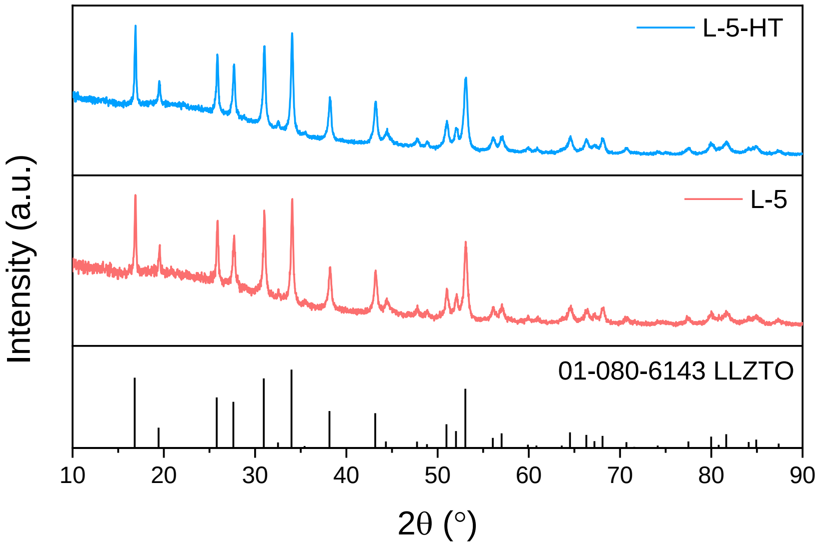

Figure S7 XRD patterns of L-5 pellets before and after heat treatment under 120 °C.

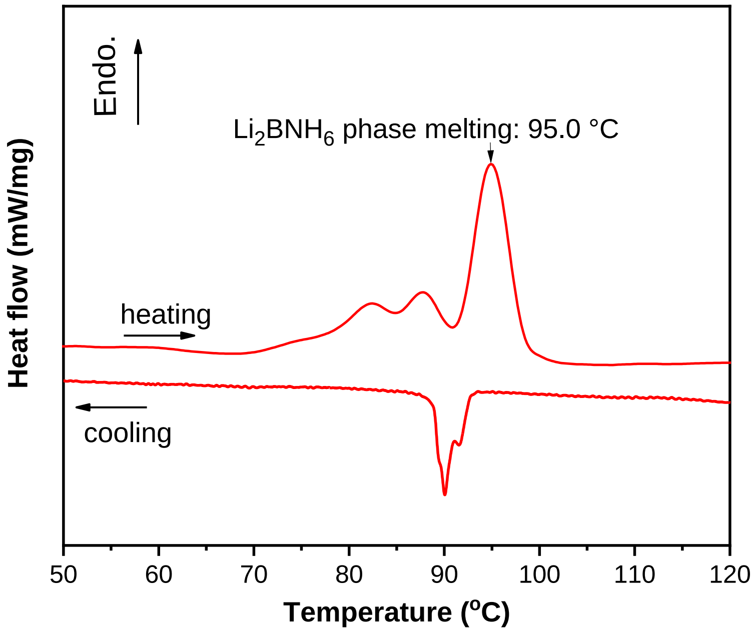

Figure S8. DSC curve of L-5 with a heating and cooling rate of 2 °C min^{-1}.

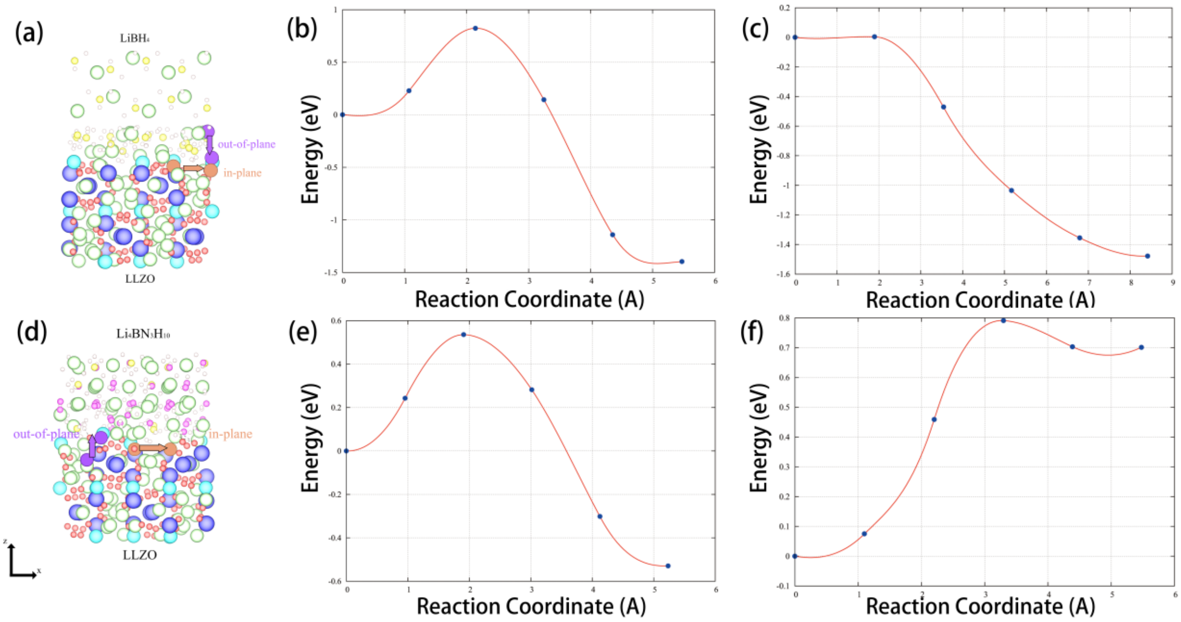

Figure S9. Schematics of in-plane and out-of-plane Li-ion migration pathways at (a) LLZO-$LiBH_4$ interface and (d) LLZO-$Li_4BN_3H_{10}$ interface. (b) and (c) CI-NEB results of in-plane and out-of-plane Li-ion migration at LLZO-$LiBH_4$ interface, respectively. (e) and (f) CI-NEB results of in-plane and out-of-plane Li-ion migration at LLZO-$Li_4BN_3H_{10}$ interface, respectively.

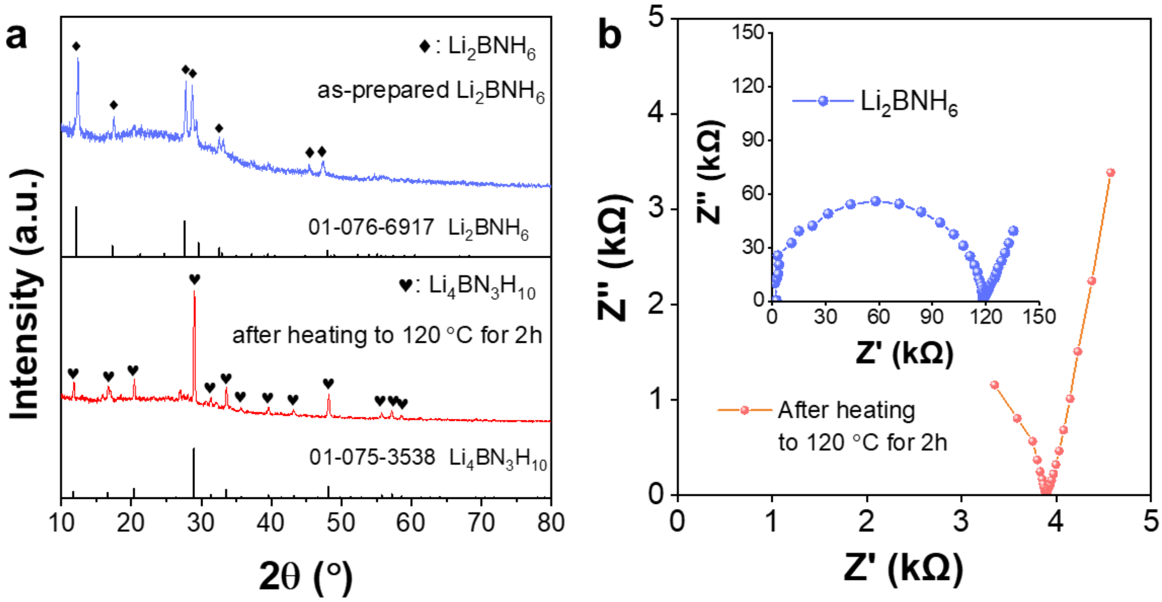

Figure S10. (a) XRD patterns of Li_2BNH_6 sample before and after heat treatment, (b) Nyquist curves of Li_2BNH_6 before and after heat treatment.

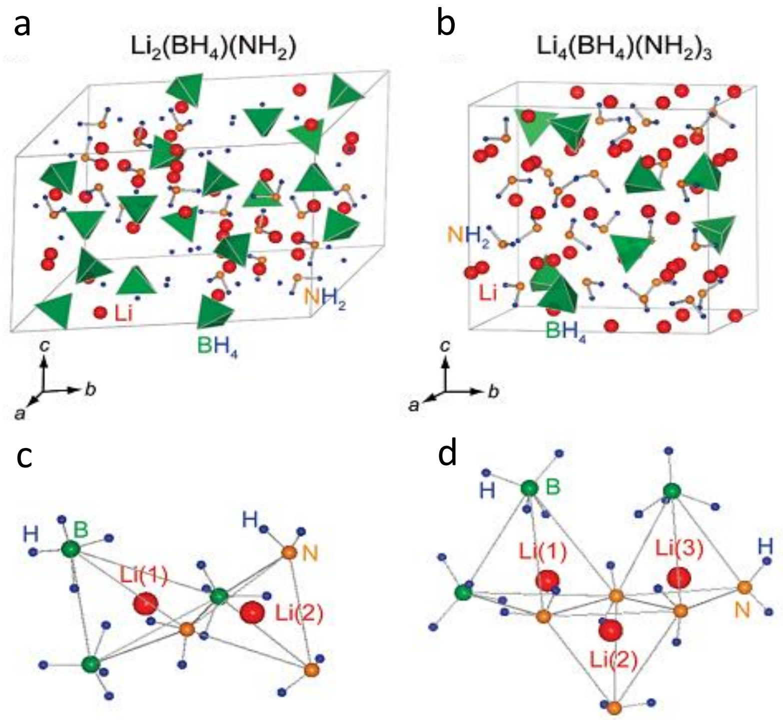

Figure S11. Crystal structures of (a) Li_2BNH_6 and (b) $Li_4BN_3H_{10}$. The green tetrahedra show the $[BH_4]^-$ anion. The Li site in the crystal structures of (c) Li_2BNH_6 and (d) $Li_4BN_3H_{10}$ (Red, green, orange and blue solid circles show Li, B, N and H, respectively).[M. Matsuo, A. Remhof, P. Martelli, R. Caputo, M. Ernst, Y. Miura, T. Sato, H. Oguchi, H. Maekawa, H. Takamura, A. Borgschulte, A. Züttel, S. I. Orimo. *J. Am. Chem. Soc.*, **2009**, *131*, 16389-16391.]

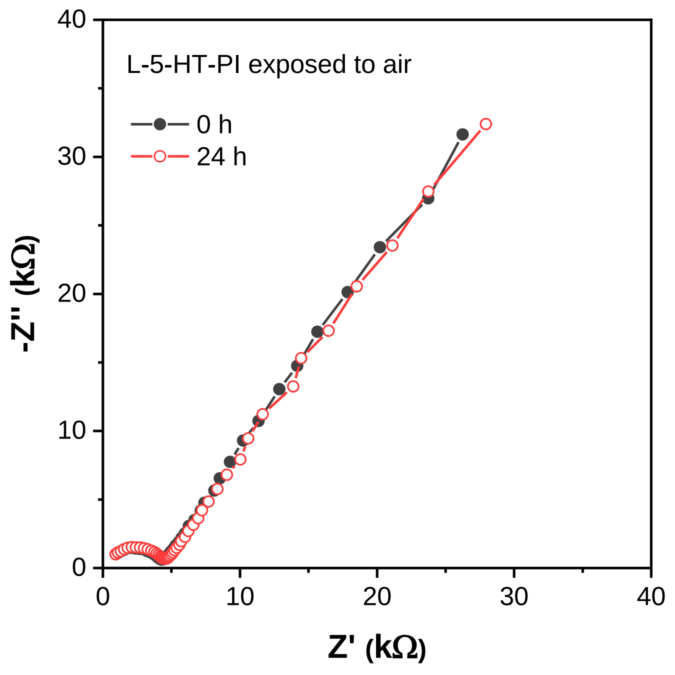

Figure S12. Nyquist curves of polyimide-coated L-5-HT pellets before and after exposing to air for 24 h.

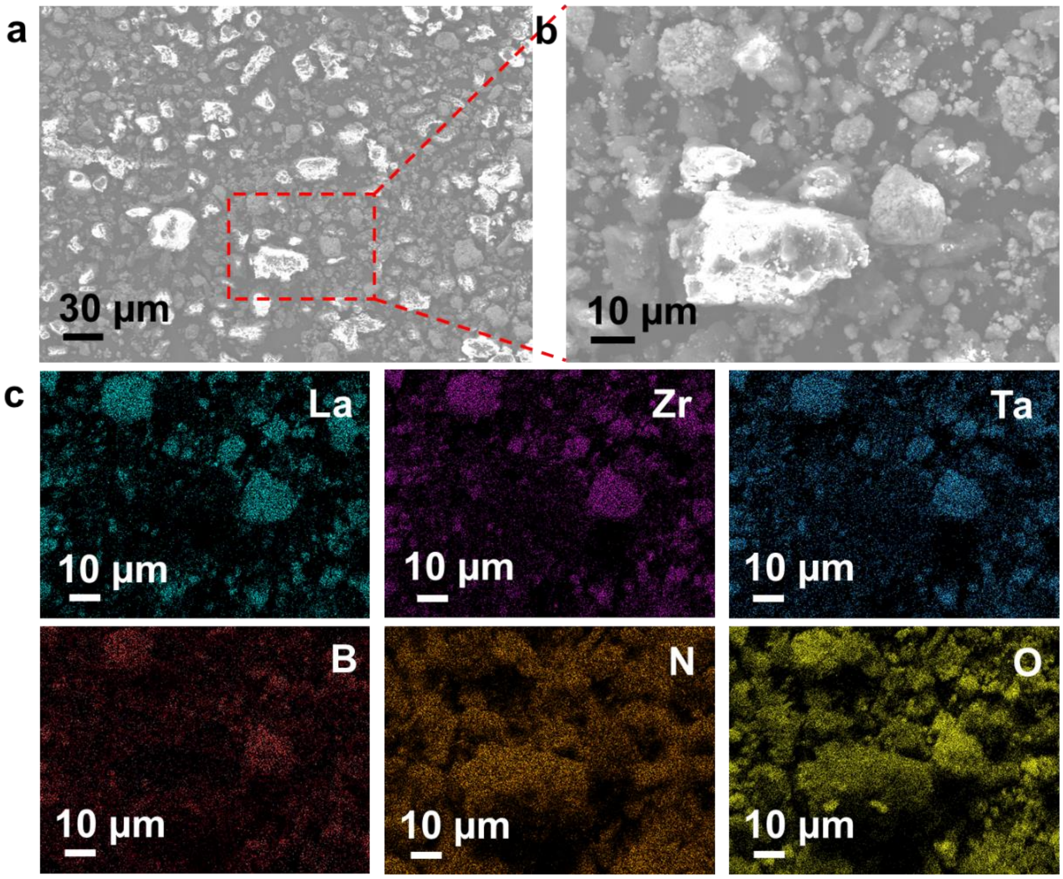

Figure S13. SEM images (a,b) and corresponding EDS mapping (c) of the L-5 sample modified with PI. The bright areas are due to the electron accumulation caused by the poor electronic conductivity of PI, indicating the surface coating of the L-5 sample particles by PI.

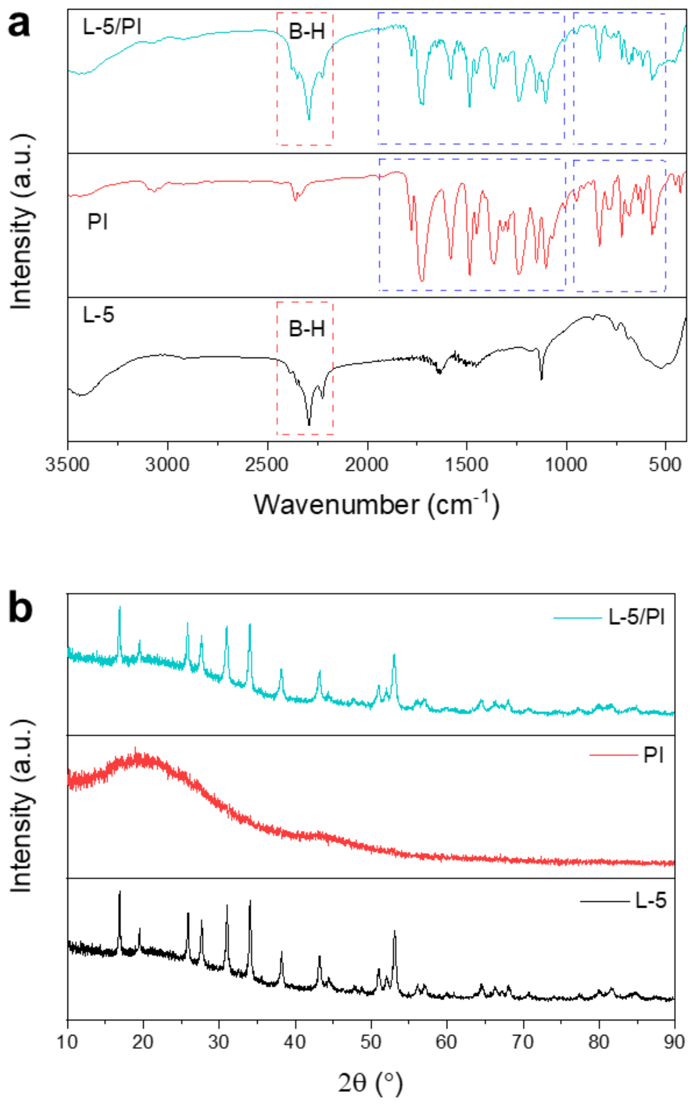

Figure S14. FTIR spectra (a) and XRD patterns (b) of the L-5 sample with and without PI.

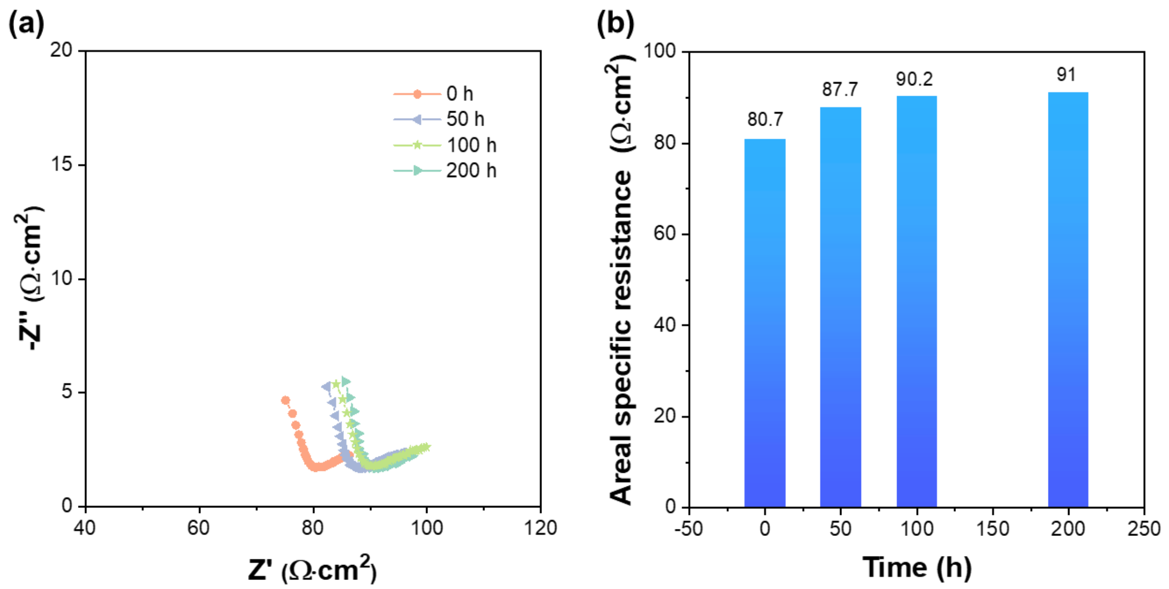

Figure S15. (a) Nyquist curves and (b) corresponding area specific resistance of L-5-HT pellets after different cycle time.

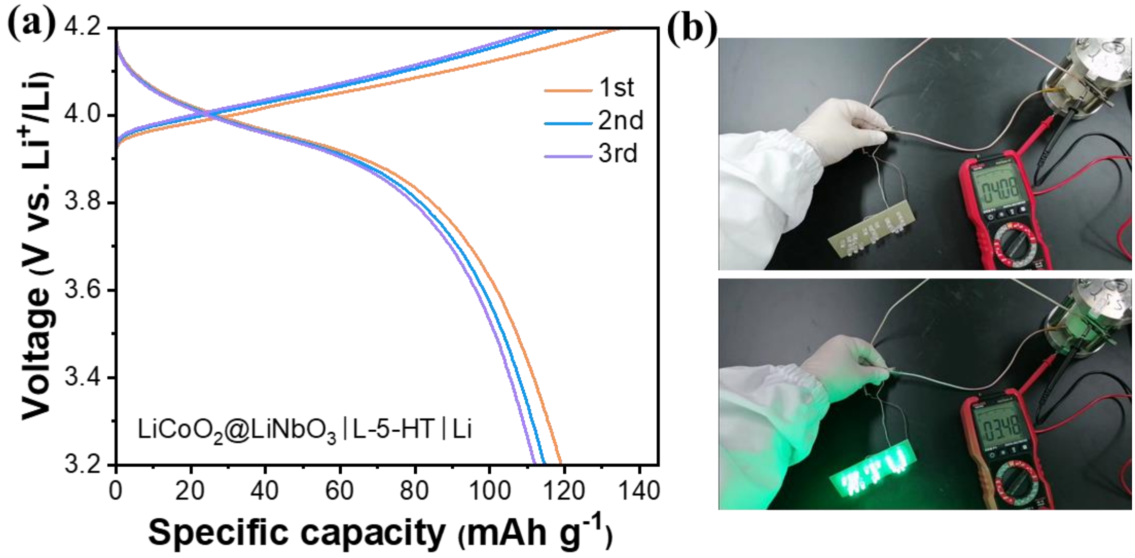

Figure S16. (a) Charge/discharge profiles of a $LiCoO_2$@$LiNbO_3$ |L-5-HT|Li full cell. (b) digital photograph of LED array powered by the full cell.
